# Association of *FKBP5* genotype with depressive symptoms in patients with coronary heart disease: a prospective study

**DOI:** 10.1007/s00702-020-02243-6

**Published:** 2020-08-29

**Authors:** Julia Brandt, Katharina Warnke, Silke Jörgens, Volker Arolt, Katja Beer, Katharina Domschke, Wilhelm Haverkamp, Stella L. Kuhlmann, Jacqueline Müller-Nordhorn, Nina Rieckmann, Kathrin Schwarte, Andreas Ströhle, Mira Tschorn, Johannes Waltenberger, Laura Grosse

**Affiliations:** 1 Department of Psychiatry and Psychotherapy, University Hospital Münster, Albert-Schweitzer-Campus 1, Geb. A9, 48149 Münster, Germany; 2 Charité-Universitätsmedizin Berlin, Corporate member of the Freie Universität Berlin, Humboldt-Universität zu Berlin, and the Berlin Institute of Health, Department of Psychiatry and Psychotherapy, Berlin, Germany; 3 Department of Psychiatry and Psychotherapy, Medical Center-University of Freiburg, Faculty of Medicine, University of Freiburg, Freiburg, Germany; 4 Charité-Universitätsmedizin Berlin, Corporate member of the Freie Universität Berlin, Humboldt-Universität zu Berlin, and the Berlin Institute of Health, Department of Internal Medicine and Cardiology, Berlin, Germany; 5 Charité-Universitätsmedizin Berlin, Corporate member of the Freie Universität Berlin, Humboldt-Universität zu Berlin, and the Berlin Institute of Health, Division of Emergency and Acute Medicine (CVK, CCM), Berlin, Germany; 6 Charité-Universitätsmedizin Berlin, Corporate member of the Freie Universität Berlin, Humboldt-Universität zu Berlin, Berlin Institute of Health, Institute of Public Health, Berlin, Germany; 7 Bavarian Food and Health Safety Authority, Oberschleißheim, Germany; 8 Social and Preventive Medicine, University of Potsdam, Potsdam, Germany; 9 Department of Cardiology, Central Hospital, Suhl, Germany; 10 Intercultural Business Psychology, Hamm-Lippstadt University of Applied Sciences, Hamm, Germany

**Keywords:** Depressive symptoms, *FKBP5*, HPA axis, CHD, Gene environment interaction, Stress-related disease

## Abstract

Depression and coronary heart disease (CHD) are prevalent and often co-occurring disorders. Both have been associated with a dysregulated stress system. As a central element of the stress system, the *FKBP5* gene has been shown to be associated with depression. In a prospective design, this study aims to investigate the association of *FKBP5* with depressive symptoms in CHD patients. *N* = 268 hospitalized CHD patients were included. Depressive symptoms were measured using the Hospital Anxiety and Depression Scale (HADS-D) at four time points (baseline, and after 1 month, 6 months, and 12 months). The functional *FKBP5* single-nucleotide polymorphism (SNP) rs1360780 was selected for genotyping. Linear regression models showed that a higher number of *FKBP5* C alleles was associated with more depressive symptoms in CHD patients both at baseline (*p* = 0.015) and at 12-months follow-up (*p* = 0.025) after adjustment for confounders. Further analyses revealed that this effect was driven by an interaction of *FKBP5* genotype with patients’ prior CHD course. Specifically, only in patients with a prior myocardial infarction or coronary revascularization, more depressive symptoms were associated with a higher number of C alleles (baseline: *p* = 0.046; 1-month: *p* = 0.026; 6-months: *p* = 0.028). Moreover, a higher number of C alleles was significantly related to a greater risk for dyslipidemia (*p* = .016). Our results point to a relevance of *FKBP5* in the association of the two stress-related diseases depression and CHD.

## Introduction

Depression and coronary heart disease (CHD) are both highly prevalent disorders and are associated with increased morbidity, mortality, and cause substantial economic costs (GBD 2013 DALYs and HALE Collaborators et al. 2015; Whiteford et al. 2013). Additionally, both medical conditions frequently co-occur (Rudisch and Nemeroff 2003). The association between CHD and depression is thought to be bidirectional (Whooley and Wong 2013). The wide range of approaches explaining this association includes a dysregulation of the hypothalamic–pituitary–adrenal (HPA) axis, which occurs in both conditions (Holsboer 2000; Jokinen and Nordstrom 2009). The HPA axis is the major stress hormone system and, in fact, exposure to stressors has repeatedly been linked with the development of both depression and cardiovascular diseases (Cohen et al. 2007; Kendler et al. 1999). There is evidence for a genetic overlap between depression and CHD, as a result of which the related (pleiotropic) genes might lead, e.g. over the shared biological pathways of a HPA axis dysregulation, to both diseases (Amare et al. 2017; McCaffery et al. 2009).

In recent years, the *FKBP5* gene has gained increased scientific interest regarding the genetic vulnerability to depression. The *FKBP5* gene codes for the FK506 binding protein 51 (*FKBP5*), a co-chaperone of the heat shock protein 90 (hsp90) that operates as an inhibitor of the glucocorticoid receptor (GR). The GR is a central element of the HPA axis and directs the body’s stress reaction by affecting gene transcription (Nicolaides et al. 2014). As part of an ultra-short negative feedback loop, the GR also induces the expression of *FKBP5*. This negative feedback restrains the activity of the HPA axis and ensures the homeostasis of the stress response (Denny et al. 2000). Within the *FKBP5* gene there is one haplotype which is described to be functional and which comprises several single nucleotide polymorphisms (SNPs) in high linkage disequilibrium, amongst others SNP rs1360780. The minor (rarer) T allele of this SNP is the high-induction allele of the *FKBP5* gene transcription and thus likely associated with a reduced cortisol binding affinity to the GR, also termed as GR resistance. A GR resistance and related prolonged glucocorticoid elevation have been shown to be associated with depression (Pariante and Lightman 2008). Accordingly, healthy T allele carriers showed a prolonged cortisol response following exposure to minor stressors (Ising et al. 2008) and less suppression of cortisol in pharmacological suppression tests (Binder et al. 2008; Touma et al. 2011).

An increasing number of studies have investigated the association of common variants in *FKBP5* and stress-related disorders like depression, posttraumatic stress disorder (PTSD), and suicidal events (Zannas et al. 2016). However, the results regarding a genetic main effect of *FKBP5* on psychiatric disorders remain inconsistent (Hernandez-Diaz et al. 2019; Rao et al. 2016; Zannas et al. 2016).

The etiology of depression is considered to be polygenetic and multifactorial (Sullivan et al. 2012). Moreover, as no specific locus for depression could be unequivocally identified yet (Major Depressive Disorder Working Group of the Psychiatric GWAS Consortium et al. 2013), gene-environment (G × E) interactions have moved into focus (Caspi et al. 2010). There is a growing body of evidence for a *FKBP5* × stressor interaction to confer risk for depression, PTSD (Wang et al. 2018), and other psychiatric phenotypes (Zannas et al. 2016). In the majority of reports, an interactive association of the *FKBP5* high induction alleles × childhood trauma with the respective phenotype was observed. Only few studies explored a *FKBP5* × E interaction regarding life stressors beyond childhood traumata and the results across the different studies are inconsistent (Zannas and Binder 2014).

In the present study, we investigated a sample with elevated risk for depression, in particular a sample of *N* = 268 hospitalized CHD patients. We aimed to (1) investigate whether *FKBP5* genotype (rs1360780) was associated with depressive symptoms in CHD patients and (2) to explore a possible G × E effect of *FKBP5* and experienced stressors on depressive symptoms. We expected that *FKBP5* genotype would be associated with depressive symptoms specifically in those CHD patients who had experienced a prior myocardial infarction (MI) or coronary revascularization entailing a more stressful CHD course. Additionally, patients were followed up and longitudinally analyzed for depressive symptoms at three time points after study inclusion (1 months, 6 months, 12 months).

## Experimental procedures

### Design and participants

**Fig. 1:**
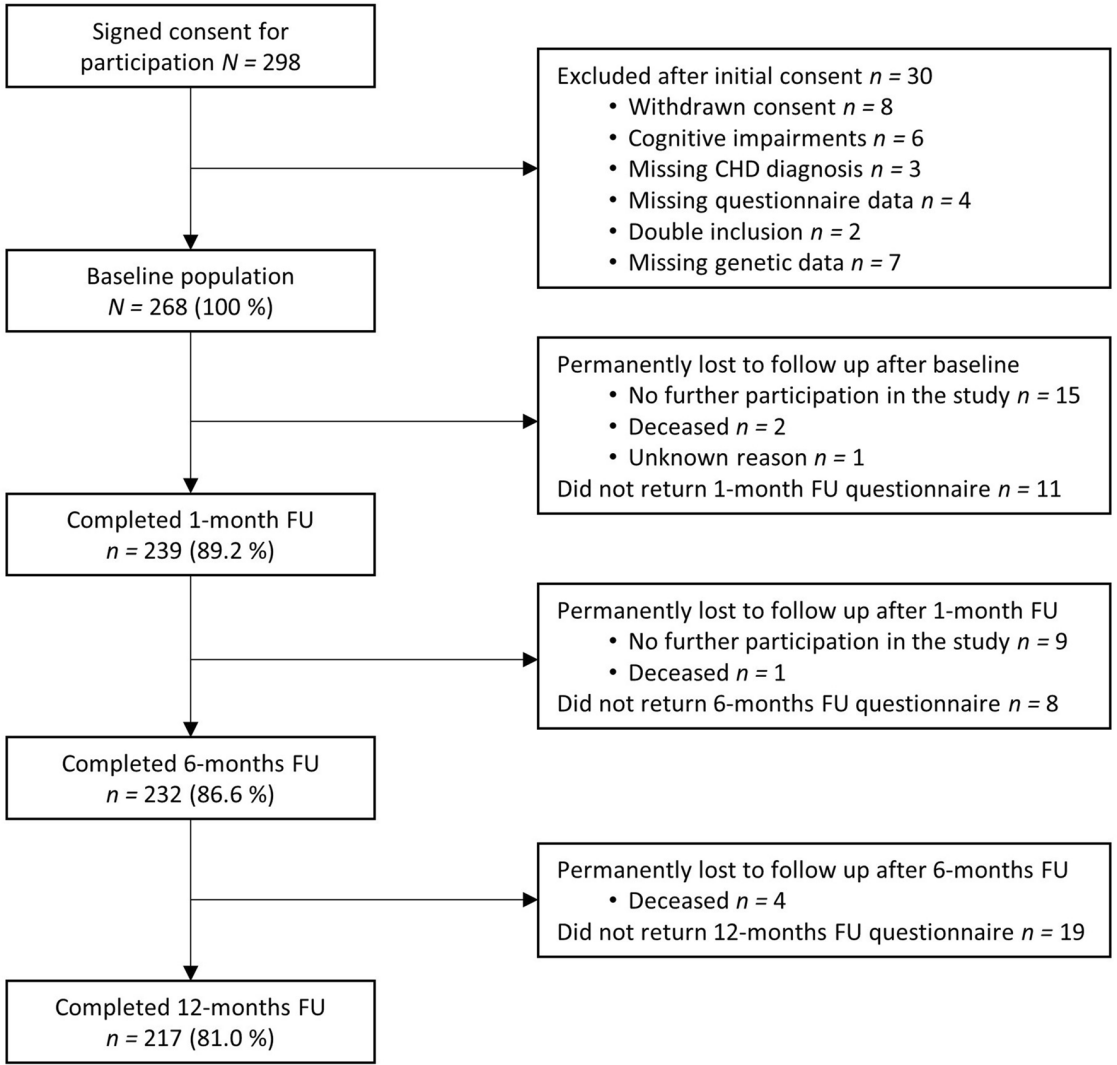
Flow chart of patient selection. *CHD* coronary heart disease, *FU* follow-up. The figure shows the selection and exclusion of study participants, response rates, and drop-outs. Participants who permanently dropped out and those who further participated in the study after not having returned one or two questionnaires are listed separately

Participants were recruited while treated at one of two recruitment sites in Münster, Germany (Department of Cardiology, University Hospital Münster) and Berlin, Germany (Department of Cardiology, Campus Virchow Clinic, Charité) between December 2012 and July 2014. We included *N* = 298 patients diagnosed with CHD and with sufficient proficiency in German (Münster site) or German or Turkish (Berlin site). Patients with a chart-documented dementia disorder, severe cognitive impairments, or with a terminal disease were excluded. The study was approved by the respective ethics committees and was in accordance with the Declaration of Helsinki. After study procedures had been fully explained, all subjects provided written informed consent. Of the *N* = 298 included subjects, *n* = 30 were excluded post hoc after inclusion because of withdrawn consent, missing CHD diagnosis, cognitive impairments, missing questionnaire data, double inclusion, or missing genetic data. Accordingly, the final sample consisted of *N* = 268 patients (Fig. 1).

The present prospective cohort study comprised several self-rate questionnaires and clinical data collection from the medical chart at baseline and a blood collection. Moreover, three follow-up assessments with questionnaires were conducted after 1, 6, and 12 months (Fig. 1).

### Clinical instruments

#### Assessment of depressive symptoms

To assess depressive symptoms the self-rated, seven-item depression subscale of the Hospital Anxiety and Depression Scale (HADS-D, German adaption) (Zigmond and Snaith 1983) was used. The HADS-D was included in the baseline assessment as well as in all follow-up questionnaires. At all time points, patients were also asked whether they received a current antidepressive treatment (antidepressant medication or psychotherapy).

#### Coronary heart disease status and related risk factors

To specify the patient’s CHD course, it was assessed whether the patient had a prior MI or coronary revascularization. Therefore, the following information was recorded from the medical chart: MI, percutaneous coronary intervention, and coronary artery bypass grafting in his/her lifetime before study inclusion. Cardiac disease severity at baseline was assessed by left ventricular ejection fraction (LVEF) which was measured by electrocardiography, magnetic resonance imaging, or cardiac catheterisation or it was abstracted from the medical chart. Moreover, we distinguished CHD patients with and without an acute coronary syndrome (ACS) at study baseline (index hospitalization). The following CHD risk factors were assessed: diabetes, body mass index (BMI), and dyslipidemia according to the medical chart as well as current smoking (determined by both patient’s self-report on the questionnaire and an additional patient interview led by the study nurse).

#### Comorbidity

The cumulative burden of physical comorbidity was assessed with the Charlson Comorbidity Index (CCI). The CCI is a measure for classifying comorbidity in longitudinal studies and predicting mortality outcomes from comorbid disease (Charlson et al. 1987). Since in our study, we were interested in somatic comorbidity other than cardiac diasease and diabetes, an important CHD risk factor (Greenland et al. 2003), we removed MI, congestive heart failure, and diabetes (which were all included in the original CCI version) from the CCI score. The CCI was used as continuous variable with higher scores indicating higher comorbidity.

### DNA extraction and SNP genotyping

We collected 20 ml of venous blood from each patient and stored it at − 20 °C until further use. The DNA of peripheral leukocytes was extracted using the FlexiGene DNA Kit (Qiagen, Hilden, Germany) in accordance with the manufacturer’s protocol.

Genotyping of subjects for *FKBP5* rs1360780 was performed using TaqMan SNP Genotyping Technology (Applied Biosystems by Life Technologies, Darmstadt, Germany) on an ABI prism 7000 Sequence Detection System. The 15 µl final reaction mix consisted of 7.5 µl TaqMan Universal PCR Master Mix, 0.375 µl Genotyping Assay (40×) containing 1 pair of target-specific primers and 1 pair of fluorescent probes, 4.125 µl water and a total of 60 ng DNA. All the assays were predesigned and validated (Applied Biosystems). DNA was amplified by polymerase chain reaction (2 min at 50 °C, 10 min at 95 °C, 15 s at 95 °C for 40 cycles, 1 min at 60 °C) in 96 well plates. For allelic discrimination the Sequence Detection Software Version 1.5.1 (Applied Biosystems) was used. For quality control, duplicate genotyping was performed in ~ 5% of the sample. No differences between duplicated analyses were observed. There was no deviation from Hardy–Weinberg equilibrium for *FKBP5* rs1360780 genotype distribution (*χ*^2^ = 0.92, *p* = 0.34).

### Statistical analyses

We used an additive model of linear regression (Binder et al. 2008), where the three genotypes were coded as 0, 1, or 2 according to the number of C alleles (TT = 0, CT = 1, and CC = 2). Associations between genotypic distribution of the *FKBP5* rs1360780 and sample characteristics were analyzed using binary logistic regression for categorical data (e.g. gender, CHD course) and linear regression for continuous data (e.g. age, CCI).

Linear regression was used to analyze both a genetic main effect and an interaction effect of *FKBP5* and the prior CHD course on self-rated depressive symptoms (HADS-D). Cronbach’s alpha was calculated to assess the internal consistency of the HADS-D scale at the different timepoints. Based on theoretical considerations, we tested the following as potential covariates in our regression analyses using forward selection: age, gender, smoking, BMI, diabetes, dyslipidemia, physical comorbidity (CCI), LVEF, and current antidepressive treatment. In all analyses, differences were regarded as statistically significant at *p ≤ *0.05. Multiple imputation was performed to deal with missing data. Details have been described previously (Kuhlmann et al. 2017). Statistical analyses were performed using IBM SPSS version 24 for Windows (SPSS Inc., Chicago, Illinois).

## Results

### Sample characteristics

Patients’ mean age at baseline was 63.5 (± 10.2) years and 80.6% of the patients were of male sex (*n* = 216; Table 1). The most prevalent *FKBP5* rs1360780 genotype was CC (*n* = 141, 52.6%), followed by the heterozygous genotype CT (*n* = 111, 41.4%) and TT homozygosity (*n* = 16, 6.0%). The minor allele frequency was 0.27, comparable to HapMap data (the HapMap minor allele frequency for rs1360780: 0.27 in European samples).

**Table 1:** Sample and medical characteristics by *FKBP5* rs1360780 genotype

	Total sample	*FKBP5* rs1360780 genotype			*p*^1^
	*N* = 268 (100%)	CC *n* = 141 (52.6%)	CT *n* = 111 (41.4%)	TT *n* = 16 (6.0%)	
Demographics					
Age *M* ± SD	63.49 ± 10.21	63.79 ± 10.33	63.40 ± 9.94	61.50 ± 11.31	0.464
Male *n* (%)	217 (81.0)	117 (83.0)	88 (79.3)	12 (75.0)	0.333
Charlson Comorbidity Index (CCI)^2^ *M* ± SD	0.81 ± 1.23	0.84 ± 1.20	0.82 ± 1.32	0.38 ± 0.89	0.318
Coronary heart disease (CHD) characteristics					
Prior MI/coronary revascularization *n* (%)	172 (64.2)	93 (66.0)	68 (61.3)	11 (68.8)	0.709
Acute coronary syndrome at baseline *n* (%)	119 (44.4)	60 (42.6)	53 (47.7)	6 (37.5)	0.761
Left ventricular ejection fraction (%) *M* ± *SD*	48.39 ± 14.12	48.25 ± 14.02	48.81 ± 14.47	46.74 ± 14.39	0.964
CHD risk factors					
Current smoking *n* (%)	70 (26.1)	39 (27.7)	28 (25.2)	3 (18.8)	0.443
Diabetes *n* (%)	72 (26.9)	37 (26.2)	29 (26.1)	6 (37.5)	0.558
Body mass index (kg/m^2^) *M* ± SD	28.36 ± 5.02	28.02 ± 4.81	28.39 ± 4.89	31.19 ± 6.85	0.059
Dyslipidemia *n* (%)	191 (71.3)	108 (76.6)	75 (67.6)	8 (50.0)	**0.016**
Antidepressive treatment					
Baseline *n* (%)	13 (4.9)	4 (2.8)	7 (6.3)	2 (12.5)	0.063
1-month *n* (%)	12 (4.5)	5 (3.5)	6 (5.4)	1 (6.3)	0.439
6-months *n* (%)	13 (4.9)	4 (2.8)	8 (7.2)	1 (6.3)	0.157
12-months *n* (%)	16 (6.0)	8 (5.7)	8 (7.2)	–	0.819

*P*-values < 0.05 were considered statistically significant and are shown in bold

*M* mean, *SD* standard deviation, *MI* myocardial infarction

^1^We used an additive model with the three genotypes coding as 0 (TT), 1 (CT), or 2 (CC) according to the number of C alleles

^2^We used a modified CCI (excluded were cardiac diagnoses and diabetes)

The variable ‘prior MI/coronary revascularization’ includes MI, percutaneous coronary intervention, and coronary artery bypass grafting before study inclusion. For analyzing categorical data, logistic regression and for analyzing continuous variables, linear regression was used. FKBP5 rs1360780 genotype was significantly associated with dyslipidemia

With regard to CHD characteristics, 64.2% (*n* = 172) of the patients had a prior MI or coronary revascularization in their lifetime before study inclusion (Table 1). At baseline, an ACS was diagnosed in 44.4% (*n* = 119) of the patients. Patients’ mean LVEF was 48.39% (± 14.12), indicating an overall mild dysfunction of the cardiac pump capacity. Regarding CHD risk factors, 26.9% (*n* = 72) patients had diabetes and 71.3% (*n* = 191) dyslipidemia. Patients’ mean BMI (28.36 ± 5.02) indicated a tendency towards overweight and *n* = 70 (26.1%) were current smokers (Table 1). Analyses revealed that a higher number of C alleles of *FKBP5* rs1360780 was significantly associated with dyslipidemia (odds ratio [OR] = 1.69, 95% confidence interval [CI] = 1.10–2.61, *p* = 0.016). No association of *FKBP5* rs1360780 with other sample characteristics, e.g. age, gender, or CHD course, was observed (Table 1). With regard to depression, patients’ initial mean HADS-D score was 5.10 (± 4.14; Table 2). The internal consistency of the questionnaire is satisfying for all timepoints (Cronbach’s alpha T0 = 0.86, T1 = 0.87, T2 = 0.87, and T3 = 0.89).

**Table 2:** Regression of depressive symptoms on *FKBP5* rs1360780 genotype

Time points	Total sample	*FKBP5* rs1360780 genotype			Linear regression analyses^1^									
		CC	CT	TT	Unadjusted		Adjusted^2^							
					*FKBP5* rs1360780		*FKBP5* rs1360780		LVEF		BMI		Anti-depressive treatment	
					*β*	*p*	*β*	*p*	*β*	*p*	*β*	*p*	*β*	*p*
Baseline	*N* = 268 (100%)	*n* = 141 (52.6%)	*n* = 111 (41.4%)	*n* = 16 (6.0%)										
HADS-D *M* ± SD	5.10 ± 4.14	5.47 ± 4.61	4.86 ± 3.57	3.56 ± 2.97	0.114	0.063	0.143	**0.015**	− 0.160	**0.007**	0.107	> 0.05	0.249	**< 0.001**
1-month	*N* = 239 (100%)	*n* = 124 (51.9%)	*n* = 101 (42.3%)	*n* = 14 (5.9%)										
HADS-D *M* ± SD	4.72 ± 3.99	4.75 ± 4.28	4.81 ± 3.84	3.71 ± 2.16	0.032	0.625	0.071	0.248	− 0.169	**0.006**	0.203	**0.001**	0.256	**< 0.001**
6-months	*N* = 232 (100%)	*n* = 124 (53.4%)	*n* = 94 (40.5%)	*n* = 14 (6.0%)										
HADS-D *M* ± SD	5.02 ± 4.23	5.01 ± 4.41	5.18 ± 4.08	4.07 ± 3.67	0.020	0.767	0.057	0.359	− 0.217	**0.001**	0.199	**0.002**	0.194	**0.002**
12-months	*N* = 217 (100%)	*n* = 113 (52.1%)	*n* = 90 (41.5%)	*n* = 14 (6.5%)										
HADS-D *M* ± SD	5.23 ± 4.52	5.72 ± 4.85	4.86 ± 4.16	3.71 ± 3.58	0.127	0.062	0.141	**0.025**	− 0.177	**0.005**	0.198	**0.002**	0.256	**< 0.001**

*P*-values < 0.05 were considered statistically significant and are shown in bold

*HADS-D* Depression subscale of the Hospital Anxiety and Depression Scale, *β* standardized beta coefficient, *M* mean, *SD* standard deviation

^1^For details regarding regression analyses, see explanation in caption of Table 1

^2^As potential covariates in the regression analyses of depressive symptoms, age, gender, smoking, body mass index (BMI), dyslipidemia, diabetes, physical comorbidity (Charlson Comorbidity Index), left ventricular ejection fraction (LVEF), and current antidepressive treatment were analyzed using forward selection. The table displays the variables which had significant covariate effects and were included in the final model

Controlling for these covariates, linear regression analyses showed that FKBP5 rs1360780 genotype significantly predicted depressive symptoms in CHD patients at baseline and at 12-months follow-up

### *FKBP5* rs1360780 genotype and depressive symptoms

Regression analyses revealed that current depressive symptoms were significantly related to *FKBP5* rs1360780 genotype, controlling for confounders (Table 2). In particular, controlling for LVEF, BMI, and antidepressive treatment, more depressive symptoms at study baseline (*p* = 0.015) and at 12-months follow-up (*p* = 0.025) were significantly associated with a higher number of C alleles.

### *FKBP5* rs1360780 genotype, depressive symptoms, and prior CHD course

**Fig. 2:**
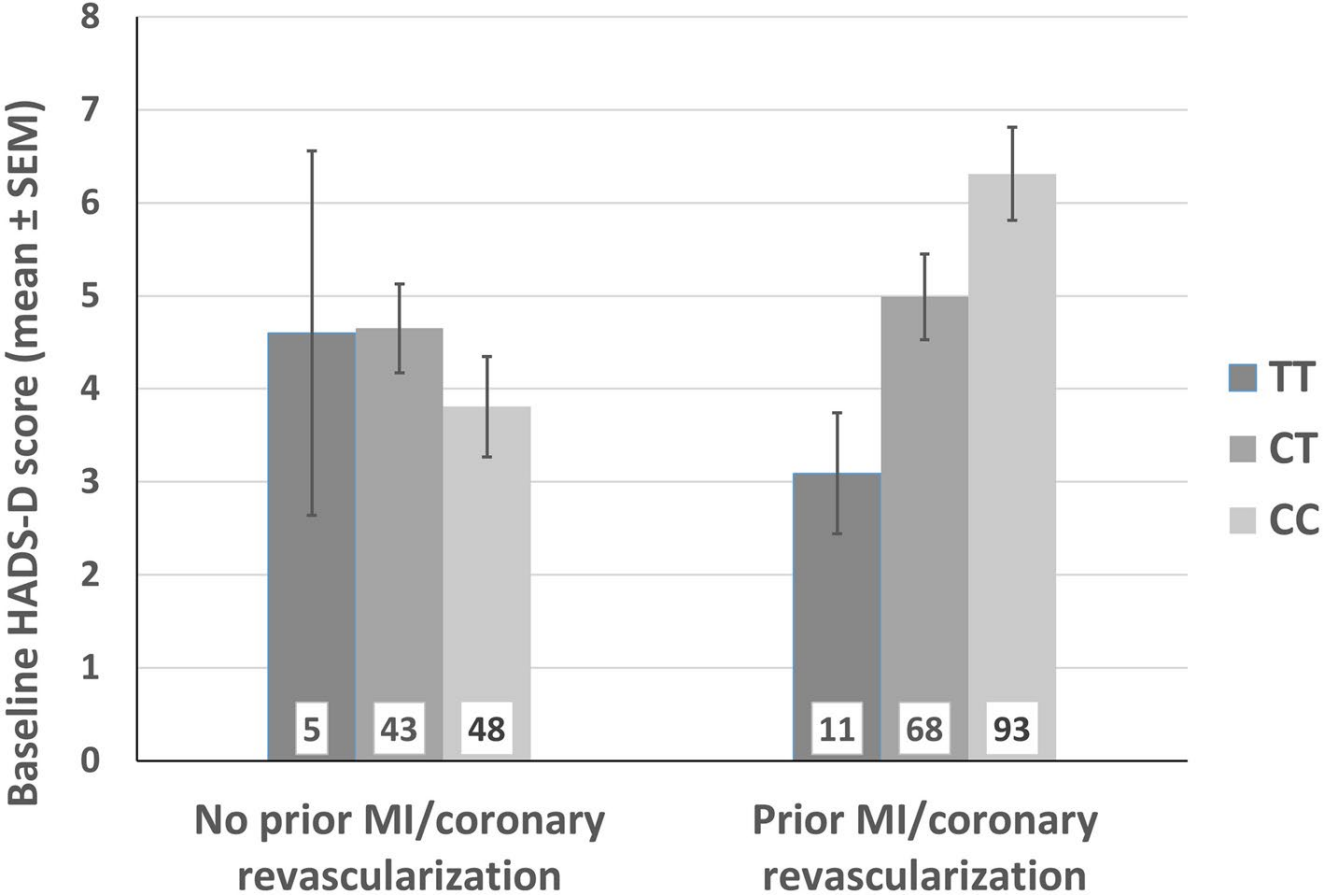
Interaction of *FKBP5* rs1360780 genotype and prior coronary heart disease (CHD) course on baseline depressive symptoms. *MI* myocardial infarction. Results indicate that a higher number of C alleles was associated with more depressive symptoms specifically in those CHD patients who have had a prior myocardial infarction or coronary revascularization (percutaneous coronary intervention or coronary artery bypass grafting). Unadjusted: *p* = 0.014, *β* = 0.442; Adjusted: *p* = 0.044, *β* = 0.356. The error bars reflect the standard error of the mean (SEM). The numbers in columns indicate the count of patients

Considering the robust evidence regarding *FKBP5* × stressor interaction on the risk for depression (Zannas and Binder 2014), we performed a linear regression model to test for possible interaction of the *FKBP5* genotype and the prior CHD course on depressive symptoms. The analyses revealed that the above reported main effect of *FKBP5* genotype on depressive symptoms (Table 2) was in fact driven by the interaction of *FKBP5* genotype × prior CHD course (Table 3). In particular, in patients who had at least one myocardial infarction or coronary revascularization before and independent of the baseline hospitalization, more depressive symptoms were associated with a higher number of C alleles (Fig. 2). Figure 2 shows the effect of the number of C alleles on baseline depressive symptoms that occurred only in those CHD patients with a prior MI or coronary revascularization. In contrast, the number of C alleles conferred no risk for depressive symptoms in CHD patients who had not experienced a CHD event before. This association was significant regarding the HADS-D scores at baseline and at 1- and 6-months follow-up (*β* = 0.439, *p* = 0.015; *β* = 0.450, *p* = 0.021; and *β* = 0.425, *p* = 0.033). After adjustment for confounders, the effect for baseline and 1- and 6-months depressive symptoms remained significant (*p* = 0.046; *p* = 0.026; *p* = 0.028) and showed a trend towards significance regarding 12-months depressive symptoms (*p* = 0.083; Table 3).

**Table 3:** Regression of depressive symptoms on the interaction between *FKBP5* rs1360780 genotype and prior coronary heart disease (CHD) course

Depressive symptoms	Included predictors						Total model	
	Genotype^1^		Prior CHD course^2^		Genotype × Prior CHD course			
	*β*	*p*	*β*	*p*	*β*	*p*	Var^3^	*p*
(A) Unadjusted models								
Baseline	− 0.093	0.364	− 0.202	0.199	0.439	**0.015**	0.047	**0.001**
1-month follow-up	− 0.178	0.113	− 0.212	0.219	0.450	**0.021**	0.037	**0.008**
6-months follow-up	− 0.180	0.108	− 0.232	0.180	0.425	**0.033**	0.020	0.057
12-months follow-up	− 0.013	0.911	− 0.196	0.269	0.296	0.142	0.014	0.108

Depressive symptoms	Included predictors						Total model	
	Genotype		Prior CHD course		Genotype × Prior CHD course			
	*β*	*p*	*β*	*p*	*β*	*p*	Var	*p*
(B) Adjusted^4^ models								
Baseline	− 0.025	0.802	− 0.161	0.300	0.353	**0.046**	0.115	**< 0.001**
1-month follow-up	− 0.124	0.246	− 0.222	0.180	0.413	**0.026**	0.145	**< 0.001**
6-months follow-up	− 0.137	0.200	− 0.262	0.114	0.414	**0.028**	0.133	**< 0.001**
12-months follow-up	− 0.011	0.916	− 0.261	0.115	0.323	0.083	0.164	**< 0.001**

*P*-values < 0.05 were considered statistically significant and are shown in bold

*β* standardized beta coefficient

^1^For details regarding regression analyses, see explanation in caption of Table 1

^2^Prior CHD course: 0 = no prior myocardial infarction (MI)/coronary revascularization (percutaneous coronary intervention or coronary artery bypass grafting), 1 =  ≥ 1 prior MI/coronary revascularization

^3^Variance explained by total model

^4^For details regarding potential covariates, see explanation in caption of Table 2

The variables left ventricular ejection fraction, body mass index, and antidepressive treatment had significant covariate effects in several analyses and were included as covariates in the respective final model

The tables show a significant effect of FKBP5 rs1360780 genotype x Prior CHD course on depressive symptoms at baseline, 1 month follow-up, and 6 months follow-up

## Discussion

In this study, we prospectively investigated relations of the *FKBP5* rs1360780 genotype with depressive symptoms in hospitalized CHD patients. The current study shows that a higher number of C alleles was associated with more depressive symptoms in CHD patients at study baseline and at 12-months follow-up after adjustment for confounders. Moreover, this study indicates that this effect is driven by an interaction of the *FKBP5* genotype with patients’ CHD course. Specifically, in patients with a prior MI or coronary revascularization, the number of C alleles was associated with depressive symptoms.

In the literature, there is some evidence for a main effect of *FKBP5* polymorphisms on depression and other psychiatric disorders (Zannas et al. 2016). A recent meta-analysis found an association of *FKBP5* rs1360780 with suicidal behavior but not with depressive disorders, whereas two other *FKBP5* SNPs were associated with depressive disorders (Hernandez-Diaz et al. 2019). Considering the central role of stressor exposure for the association of *FKBP5* and psychiatric disorders (Wang et al. 2018; Zannas et al. 2016), our findings point in the direction that, as a chronic disease, CHD in itself might act as stressor. Regarding other stress-responsive genes of the HPA axis and their role in the bidirectional association between CHD and depression, the following explanation has recently been proposed: the organism might consider CHD (respectively depression) as chronic stress and might induce functional regulatory changes in the stress-responsive genes of the HPA axis. The subsequent HPA axis dysregulation might increase the risk for depressive symptoms (respectively CHD) (Amare et al. 2017). Another interpretation might be that *FKBP5* is a pleiotropic gene increasing the risk for both depression and CHD. This is supported by our findings, that a higher number of C alleles was not only associated with more depressive symptoms but also with higher risk for dyslipidemia, an important CHD risk factor (Greenland et al. 2003). Recent findings of a genetic overlap between mood disorders and cardiovascular disease included various genes which regulate the HPA axis (Amare et al. 2017). Since we found an association with both diseases, *FKBP5*, a central element of the HPA axis, might also be an interesting target gene of such investigations. To date, there is only few evidence on a possible association of CHD or CHD risk factors with *FKBP5*. As CHD is robustly linked to peripheral inflammation (Kaptoge et al. 2014), and *FKBP5* promotes inflammation by activating the central immune regulator NF-κB (Erlejman et al. 2014), it is plausible to assume that *FKBP5* shapes cardiovaskular risk via this pathway. A recent study has shown that aging in combination with stress (childhood trauma or depressive phentotypes) contributes to an epigenetic up-regulation of *FKBP5 (*Zannas et al. 2019*)*. These epigenetic changes were associated with increased NF-κB-related inflammation and history of myocardial infarction. In further studies a link between *FKBP5* and insulin resistance, triglyceridemia, obesity, and diabetes (Fichna et al. 2016; Gragnoli 2014; Ortiz et al. 2018) has been described. The exact role of *FKBP5* in the risk for CHD and in the association of CHD with depression as well as the underlying pathophysiological mechanisms should be analyzed in further studies.

It should be taken into account that, with a mean age of 63.5, our sample is one of elderly people. Regarding the above mentioned complex relationship between *FKBP5*, stress, aging, and inflammation, there is another interesting aspect: A previous review suggests that perfusion deficits in the elderly might trigger microglial activation and subsequent neuroinflammation which in turn plays a central role in the pathophysiology of late-life depression (Popa-Wagner et al. 2014). Considering the association between a HPA axis hyperactivity and the accompanying glucocorticoid resistance with inflammation (Zunszain et al. 2011), it can be assumed that *FKBP5* might be associated with neuroinflammation following perfusion deficits in the elderly. However, further studies would have to investigate a possible role of *FKBP5* in this context.

With regard to investigating an elderly sample, it is also important to consider that there is evidence for age specific alterations of the HPA axis itself (Deuschle et al. 1997). A systematic review investigating the HPA axis and aging in depression reports a high degree of HPA axis dysregulation in depressed older adults with differences compared with younger adults (Belvederi Murri et al. 2014). The authors proposed that this might depend on several mechanisms, including physical illnesses and alterations in the central nervous system (CNS) (Belvederi Murri et al. 2014). Regarding the CNS, it has been shown that chronic unremitting stress and the subsequent stimulation of the HPA axis in older adults often results in neuronal degeneration especially in hippocampal neuron loss (Sapolsky 1999). As a negative-feedback mediator of glucocorticoid secretion the hippocampus itself is essential for an effective stress response (Sapolsky 1999). Regarding the CNS in late life depression, another hypothesis has emerged in recent years: The concept of ‘brain reserve’ describes that some individuals might have an increased ‘baseline adaptive neuroplasticity’, providing greater dynamic capacity for adjusting and remodeling cortical circuits to various stressors (Freret et al. 2015). Regarding a possible role of *FKBP5* in the concept of ‘brain reserve’ in late life depression there are two hypothesis: first, one could assume that considering the adverse effects of a dysregulated HPA axis on the brain structure, *FKBP5*, as a central element of the HPA axis, might modulate the ‘brain reserve’ itself and gains its influence on depressive disorders via this pathway. Second, it is conceivable that the individual’s ‘brain reserve’ is an independent neuroprotective factor and makes the organism more or less vulnerable to *FKBP5*-mediated HPA axis dysregulation and its adverse effect. Considering the ‘brain reserve’ as an individual and in its extent varying resilience factor, it could contribute to the inconsistent findings regarding a genetic effect of *FKBP5* on depressive disorders. Regarding *FKBP5* and its association with pathophysiological alterations in the CNS in depressive disorders, further studies are needed.

Our finding, of carriers of the *FKBP5* C allele with a more stressful CHD course displaying higher depression scores, complements the literature on the importance of *FKBP5* × environment interaction. Whereas the majority of studies investigated the G × E interaction with regard to the environmental factor of childhood trauma, there is only a limited number of heterogenous studies exploring stressors in adult life (Zannas et al. 2016). In the latter regard, associations of the *FKBP5* minor alleles with depression and anxiety scores in patients with advanced gastric cancer (Kang et al. 2012) and with depressive symptoms in kidney transplant recipients (Shinozaki et al. 2011) were reported. Furthermore, rs1360780 T allele carriers who were exposed to adult stress showed greater risk for long-term negative health implications (Lessard and Holman 2014). Interestingly, a recent study oberved that rs1360780 T allele carriers experienced greater post traumatic growth (PTG) following exposure to the hurricane Katrina compared to subjects with the CC genotype (Dunn et al. 2014). Other studies, however, did not find an association of *FKBP5* × adult adverse life event on risk for PTSD or depression, respectively (Binder et al. 2008; Lahti et al. 2015; Lavebratt et al. 2010).

Interestingly, in the present study a higher number of the major C allele was found to be associated with more depressive symptoms in the sample of CHD patients, while the majority of previous studies found the minor T allele to confer risk of mood and anxiety disorders (Hernandez-Diaz et al. 2019; Zannas et al. 2016). However, in line with the present findings, several studies reported an association of the major alleles of *FKBP5* SNPs with depression (Zobel et al. 2010), suicidal events in depressed patients (Brent et al. 2010), and more depressive symptoms in male adolescents in the context of victimization (VanZomeren-Dohm et al. 2015). The complex nature of the *FKBP5*-mediated stress response, especially when taking different stressors into account, is also apparent from two studies investigating *FKBP5 *× childhood trauma: whereas homozygous rs1360780 T allele carriers who experienced a traumatic childhood event were at the greatest risk for depression or PTSD, respectively, non-traumatized TT homozygotes were at lower risk compared to C allele carriers (Xie et al. 2010; Zimmermann et al. 2011). In this context, it has previously been suggested that the term ‘risk gene’ should be abandoned in favor of ‘plasticity gene’ rendering a person more vs. less sensitive to the environment (Belsky and Hartman 2014). The hypothesis of potentially beneficial effects of a hyper-responsive HPA axis is supported by another, above mentioned, study describing greater PTG in T allele carriers compared to subjects with the CC genotype after they experienced the hurricane Katrina (Dunn et al. 2014). These data are in accordance with our results as we could show that, specifically in patients with a prior MI or coronary revascularization, the T allele was associated with less depressive symptoms in our CHD sample.

Inconsistencies across the different studies regarding the direction of allelic association may furthermore be due to differences of the investigated stressors regarding time of exposure, duration, severity, and type (psychological vs. primarily somatic, acute vs. chronic).

In this regard, we could show that baseline ACS x *FKBP5* interaction did not significantly predict depressive symptoms at the different time points. Our findings that a prior more stressful CHD course, in contrast, interacts with *FKBP5* to predict depressive symptoms after hospitalization due to CHD, might be explainable by a required latency between the exposure to the CHD-related stressor and patient’s depressiveness due to delayed pathophysiological alterations. Moreover, it is conceivable that only after exposure to the initial CHD-related stressor (and the assumed subsequent HPA axis dysregulation), repeated, even lower stress like the hospitalization might interact with *FKBP5* on the risk for depressiveness. Accordingly, significant variability in HPA response patterns has been reported among patients with depression possibly due to different stressor characteristics such as type of stressor and duration (Burke et al. 2005; Dickerson and Kemeny 2004). The focus of previous studies investigating the *FKBP5* × environment interaction was mainly the distinction into adult and childhood trauma, and specificities of the latter (Zannas et al. 2016). Our findings suggest that the exact characteristics of stressors occuring in adulthood might be central for the *FKBP5 *× stressor interaction on risk for depression or possibly also other stress-related psychiatric disorders.

Moreover, our results indicate a long-term effect of *FKBP5* on patients’ depressive symptoms, as *FKBP5* was not only associated with patients’ depressive symptoms directly after hospitalization but also after 12 months. The *FKBP5* × CHD course interaction predicted depressive symptoms in the first 6 months after hospitalization, whereas it was only trendwise associated with depressive symptoms after 12 months. Those results indicate that the prior CHD course might be especially relevant for the relation of *FKBP5* with depressive symptoms in the period following the hospitalization due to CHD.

A number of limitations need to be considered when interpreting the present results: The sample size is small and particularly only a few patients were homozygous for the rarer T allele. Moreover, generalizability is limited to subsyndromal depression symptoms. Referring to CHD and depressive disorders as both phenotypic heterogeneous and chronic diseases, further limitations have to be mentioned: First, although we tried to specify patients’ CHD, it is still a simplistic approach regarding such a heterogenous disease, in particular, as we could show the importance of the CHD course in our present analyses. A control group without CHD would have been informative regarding the relevance of CHD for the association of *FKBP5* and depressive symptoms. Second, as another central limitation, it has to be noted that episodes of depressive disorders in the patients’ history were not considered. Our study showed a link between CHD and depressive symptoms depending on *FKBP5* genotype, but nonetheless, information on the temporal course of both chronic diseases might be helpful to further investigate potential underlying mechanisms and lead to a more differentiated view.

Though we did not query the patient’s ethnicity, the majority of patients indicated to have parents who were both born in Germany (78%) or countries where the majority of inhabitants are also European Caucasians at the time of birth (17%). Finally, we were not able to control for some additional factors that have been shown to be modulate the influence of *FKBP5* gene variation on psychiatric disorders e.g. trauma, especially in childhood, or comorbidity with other psychiatric disorders (Zannas and Binder 2014).

To our knowledge, this is the first study investigating the association of *FKBP5* gene variation with depressive symptoms in the context of CHD. Overall, our study points to a relevance of *FKBP5* genotype in conferring depressive symptoms in CHD patients, particularly in those patients with a prior MI or coronary revascularization. Moreover, the study indicates that *FKBP5* might confer a shared genetic risk for both CHD and depression. Further research will have to unravel the nature of *FKBP5* × adult stress interactions and to investigate whether determining *FKBP5* genotype might aid in identifying CHD patients at risk for categorical clinical depression. This could allow for a transdiagnostic personalized medicine approach considering both cardiac and psychiatric aspects in an attempt to alleviate the individual disease burden by offering early preventive or therapeutic option for patients at risk.

## Data Availability

The authors confirm that the data supporting the findings of this study are available within the article.

## Code availability

Statistical analyses were performed using IBM SPSS version 24 for Windows (SPSS Inc., Chicago, Illinois).
